# Worldwide patterns of haplotype diversity at 9p21.3, a locus associated with type 2 diabetes and coronary heart disease

**DOI:** 10.1186/gm51

**Published:** 2009-05-12

**Authors:** Kaisa Silander, Hua Tang, Sean Myles, Eveliina Jakkula, Nicholas J Timpson, Luigi Cavalli-Sforza, Leena Peltonen

**Affiliations:** 1 Institute of Molecular Medicine FIMM, University of Helsinki, and Unit of Public Health Genomics, National Institute for Health and Welfare, Tukholmankatu 8, 00290 Helsinki, Finland; 2 Department of Genetics, Stanford University School of Medicine, 300 Pasteur Drive, Stanford, CA 94305, USA; 3 Institute for Genomic Diversity, Cornell University, Ithaca, NY 14853-2703, USA; 4 Wellcome Trust Sanger Institute, Wellcome Trust Genome Campus, Hinxton, Cambridge, CB10 1SA, UK; 5 MRC CAiTE Centre, University of Bristol, Oakfield Grove, Clifton, Bristol BS8 2BN, UK

## Abstract

A 100 kb region on 9p21.3 harbors two major disease susceptibility loci: one for type 2 diabetes (T2D) and one for coronary heart disease (CHD). The single nucleotide polymorphisms (SNPs) associated with these two diseases in Europeans reside on two adjacent haplotype blocks with independent effects on disease. To help delimit the regions that likely harbor the disease-causing variants in populations of non-European origin, we studied the haplotype diversity and allelic history of the 9p21.3 region using 938 unrelated individuals from 51 populations (Human Genome Diversity Panel). We used SNP data from Illumina's 650Y SNP arrays supplemented with five additional SNPs within the region of interest. Haplotype frequencies were analyzed with the EM algorithm implemented in PLINK. For the T2D locus, the TT risk haplotype of SNPs rs10811661 and rs10757283 was present at similar frequencies in all global populations, while a shared 6-SNP haplotype that carries the protective C allele of rs10811661 was found at a frequency of 2.9% in Africans and 41.3% in East Asians and was associated with low haplotype diversity. For the CHD locus, all populations shared a core risk haplotype spanning >17.5 kb, which shows dramatic increase in frequency between African (11.5%) and Middle Eastern (63.7%) populations. Interestingly, two SNPs (rs2891168 and rs10757278) tagging this CHD risk haplotype are most strongly associated with CHD disease status according to independent clinical fine-mapping studies. The large variation in linkage disequilibrium patterns identified between the populations demonstrates the importance of allelic background data when selecting SNPs for replication in global populations. Intriguingly, the protective allele for T2D and the risk allele for CHD show an increase in frequency in non-Africans compared to Africans, implying different population histories for these two adjacent disease loci.

## Findings

A 100 kb region on chromosome 9p21.3 has been recently identified as harboring susceptibility variants to both coronary heart disease (CHD)/myocardial infarction [1-5] and to type 2 diabetes (T2D) [6,7] in study populations of European origin. The associated variants reside on two adjacent haplotype blocks, as defined using HapMap data from 30 trios of Northern and Western European ancestry (CEU) collected by the Centre d'Etude du Polymorphisme Humain (CEPH) [8]. The effects of the variants are independent: the T2D risk variant does not confer increased risk of CHD, and vice versa [9,10]. The variants associated with CHD also contribute to the risk of other disease phenotypes, such as abdominal aortic and intracranial aneurysms [10] and ischemic stroke [11]. The extensive linkage disequilibrium (LD) seen in this genomic region in the CEU HapMap population is diminished in the other HapMap populations of African, Chinese and Japanese ancestry, both for pair-wise LD levels as well as size of the haplotype blocks. In addition, the disease-associated variants are located in a genomic region of unknown function. These two issues contribute to the challenge of identifying the possible causative variants, and studying their effects in populations of non-European ancestry.

To help delimit the regions that likely harbor the disease-causing variants in populations of non-European origin, we studied the haplotype diversity and allelic history of the 9p21.3 region using existing genotype data from 938 unrelated individuals from 51 populations, from Sub-Saharan Africa, North Africa, Europe, the Middle East, South/Central Asia, East Asia, Oceania and the Americas (the Human Genome Diversity Panel (HGDP-CEPH) [12]). Descriptions of genome-wide single nucleotide polymorphism (SNP) variation across these populations have recently been published by two independent groups, using Illumina's HumanHap550 BeadChip in 29 populations [13] and HumanHap650Y BeadChip in 51 populations [14]. Here we present an analysis of the 650Y SNP data [14], supplemented with five additional SNPs: rs11790231, rs10965227, rs7045889 and rs10811661 typed using Sequenom iPLEX chemistry (Sequenom, San Diego, CA, USA) and rs1333049 typed using KASPar chemistry (Kbioscience, Hoddesdon, Herts, UK) [15]. The additional SNPs were selected to include disease-associated SNPs and haplotype-tagging SNPs that were not present on the 650Y chip. Genotype quality controls included eight duplicates of a CEPH sample and eight water controls in every 384-well plate. Genotype clusters were manually reviewed and genotyping success rate for each SNP was >98.9%. The genotype data included the variants associated with T2D (rs10811661 and rs10757283), as well as eight variants associated with CHD or found in high LD (r^2 ^> 0.9) with associated variants across a 44 kb region (rs10116277, rs1537370, rs10738607, rs4977574, rs944797, rs2383207, rs1537375 and rs1333049) in the HapMap CEU population. Haplotype frequencies were analyzed with the EM algorithm implemented in PLINK [16], which estimates the frequencies of probabilistically inferred sets of haplotypes within a population-based sample set. Haplotype structure was visualized with Haploview [17]. The analysis was performed for each geographic region separately, including in the analysis all individuals from the various populations in that geographic region. Due to the small number of unrelated individuals studied here and the uncertainty of phasing, we omit region-specific haplotypes with frequencies <5%.

**Figure 1:**
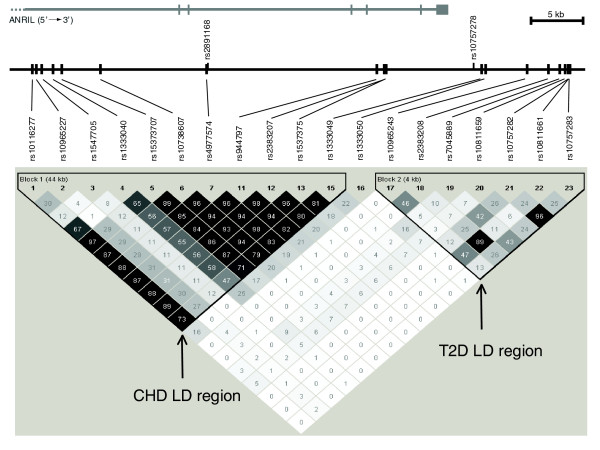
**The pattern of LD in the European HGDP samples on chromosome 9:22071397-22124172, a region approximately 53 kb long**. R^2 ^values between each SNP pair are shown in shades of grey (black R^2 ^= 1, white R^2 ^= 0) and within each box. The CHD and T2D LD regions in Europeans are clearly separate. The SNPs best tagging the disease-associating haplotypes (rs4977574 and rs10811661) are in bold-face. The positions of two SNPs that have been identified as most strongly associated with CHD in two separate fine-mapping studies of Europeans, rs2891168 and rs10757278 (see text), are shown above the genomic sequence line. The position of the *ANRIL *gene is shown at the top, while the *CDKN2B *gene is located 72 kb upstream of the first SNP shown, rs10116277.

Our analyses show that the T2D and CHD loci have different allelic histories, which is in agreement with their independent effects on disease. The haplotype structure of the critical region containing the CHD- and T2D-associated SNPs in the HGDP European sample is shown in Figure 1, and for comparison the same region is shown in the HGDP African sample (Additional data file 1). For the T2D locus, the T allele of rs10811661 was found to be associated with disease risk [6], while a larger meta-analysis identified haplotype TT of rs10811661 and rs10757283 as most strongly associated with disease [7]. The TT risk haplotype is present in similar frequencies in all global populations, while a shared 6-SNP haplotype that carries the protective C allele of rs10811661 is found at a frequency of 2.9% in Africans and 41.3% in East Asians and is associated with low haplotype diversity (Table 1). This frequency difference between populations and lack of haplotype diversity of the protective allele is reminiscent of the TCF7L2 T2D locus, in which the protective allele is found at a frequency of 10 to 31% in Africans but at 95% in East Asians [15,18]. Such large allele frequency differences and lack of haplotype diversity are indications of the past action of positive natural selection [19]. However, the degree of population differentiation for rs10811661 is not unusual compared to random SNPs in the genome (Fst = 0.126 (*P *= 0.224) across the 51 HGDP populations) [15], suggesting a neutrally selected region, while the protective allele of rs7901695 at the TCF7L2 locus was likely driven to high frequency in East Asians (global Fst = 0.213 (*P *= 0.08) across the 51 HGDP populations) by positive selection [18].

**Table 1:** The frequencies of estimated 6-SNP haplotypes^† ^for the 9p21.3 T2D locus in seven different geographic regions

rs2383208	rs7045889	rs10811659	rs10757282	rs10811661	rs10757283	Africa N = 102	Middle East N = 160	Europe N = 156	C.S. Asia N = 200	E. Asia N = 229	Oceania N = 28	America N = 63
***G***	***A***	***A***	***G***	***C***	***T***	0.029	0.197	0.157	0.125	0.393	0.446	0.167
A	A	A	G	T*	T*	0.118	0.187	0.266	0.221	0.215	0.196	0.206
G	G	A	A	T*	T*	0.131	0.022	NF	NF	NF	NF	NF
A	A	A	A	T*	T*	0.182	NF	NF	NF	NF	NF	NF
G	A	A	A	T*	T*	0.052	NF	NF	NF	NF	NF	NF
A	G	A	A	T	C	0.302	0.334	0.308	0.418	0.244	0.089	0.547
A	A	G	A	T	C	0.022	0.177	0.198	0.199	0.091	0.161	NF
A	G	G	A	T	C	0.051	0.038	0.046	0.018	0.011	0.071	0.024
G	G	A	A	T	C	0.042	0.015	NF	NF	NF	NF	NF
A	A	A	A	T	C	0.053	NF	NF	NF	NF	NF	0.024

The TT haplotype of SNPs rs10811661 and rs10757283 is the T2D risk haplotype and is in bold-face and marked with an asterisk, while the protective haplotype (GAAGCT) is in bold-face and italicized. ^†^Population-specific haplotypes <5% frequent were omitted. NF, haplotype not found in given geographic region.

**Figure 2:**
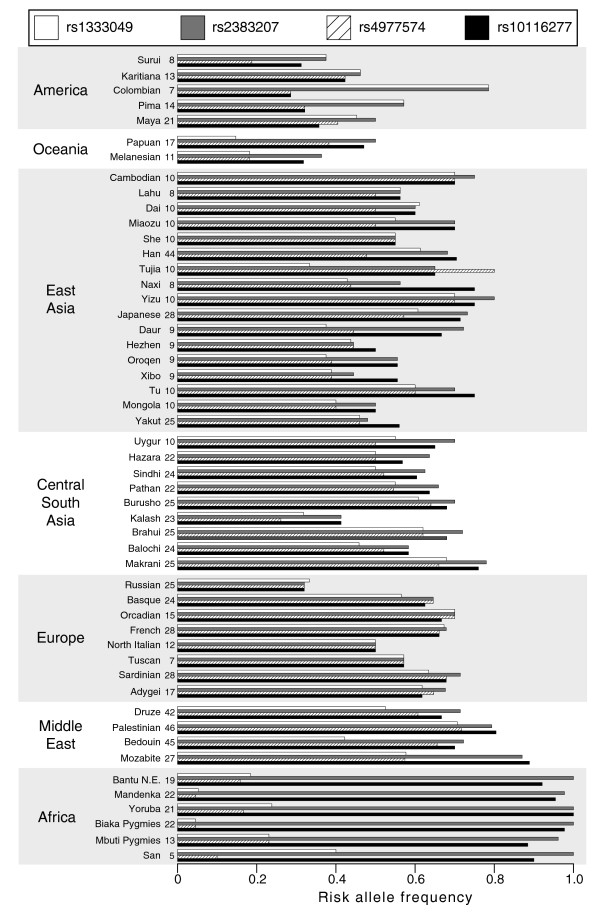
**Risk allele frequencies across 51 populations for four CHD-associated SNPs that are highly correlated in European populations**. The number of individuals in each population is provided to the right of each population name.

The risk allele frequencies of four of the CHD-associated SNPs are shown in Figure 2. Although these SNPs show highly similar allele frequencies and are in almost perfect LD in European populations (r^2 ^> 0.9), they show dramatic differences in allele frequencies across other populations, most notably in African populations. In order to decipher which of these risk alleles might be the true causative variant (or in high LD with it) and thus may be suitable for testing in non-European populations, we studied the haplotype diversity across the different geographic regions for eight highly correlated CHD-associated SNPs (r^2 ^> 0.9 in CEU HapMap population; Table 2). All populations appear to share a core risk haplotype as a part of the longer risk haplotype identified in Europeans. This risk haplotype (GGGC, for SNPs rs4977574, rs944797, rs2383207, rs1537375) spans >17.5 kb, and is tagged by the risk allele G of SNP rs4977574. The G allele of rs4977574 is also the best tag SNP for the longer risk haplotype (>44.1 kb) that is most common in all populations. All the other CHD-associated risk alleles were also found on other haplotypes in non-European populations. The risk allele of rs4977574 shows a dramatic change in frequency between African and Middle Eastern populations (Figure 2), and tags the only 8-SNP haplotype of African origin that becomes common in European populations (Table 2). Interestingly, two comprehensive fine-mapping studies of this region in case-control samples have identified SNPs in the same haplotype block as rs4977574 (rs2891168 and rs10757278, shown in Figure 1) as most strongly associated with disease [1,9]. These three SNPs (rs4977574, rs2891168, and rs10757278) are highly correlated with each other in all four HapMap populations and are the most appropriate for further analyses in non-Europeans. The 44 kb LD region harbors the *ANRIL *(antisense noncoding RNA in the INK4 locus) gene, which codes for a large antisense non-coding RNA, and was found to be expressed in tissues involved in atherosclerosis [9]. The three CHD risk haplotype tagging SNPs are located in regions of regulatory potential, as defined from alignments of several mammalian sequences [20,21], and thus may be representing the actual functional domains associated with disease risk.

**Table 2:** The frequencies of estimated haplotypes^† ^for eight CHD-associated SNPs in seven different geographic regions

rs10116277	rs1537370	rs10738607	rs4977574	rs944797	rs2383207	rs1537375	rs1333049	Africa N = 102	Middle East N = 160	Europe N = 156	C.S. Asia N = 200	E. Asia N = 229	Oceania N = 28	America N = 63
G	C	T	A	A	A	T	G	NF	0.209	0.384	0.327	0.328	0.554	0.449
***T***	***T***	***C***	G*	G*	G*	C*	***C***	0.115	0.516	0.536	0.478	0.470	0.161	0.302
***T***	***T***	***C***	G*	G*	G*	C*	G	NF	0.100	0.031	0.029	NF	0.143	NF
G	C	***C***	G*	G*	G*	C*	***C***	NF	0.021	0.017	NF	0.019	NF	0.032
***T***	***T***	***C***	A	***G***	***G***	***C***	G	0.021	0.019	NF	NF	NF	NF	NF
***T***	***T***	T	A	A	***G***	***C***	G	0.345	0.041	NF	NF	NF	0.054	NF
***T***	C	T	A	A	***G***	***C***	G	0.246	0.013	NF	NF	NF	NF	0.016
***T***	***T***	***C***	A	A	***G***	***C***	G	NF	NF	NF	0,035	0.092	0.054	NF
***T***	***T***	T	A	***G***	***G***	T	G	0.129	0.031	NF	0,027	NF	NF	NF
G	C	T	A	***G***	***G***	***C***	***C***	NF	NF	NF	NF	NF	NF	0.147
G	C	T	A	A	***G***	***C***	G	0.027	NF	NF	NF	NF	0.036	NF
***T***	***T***	T	A	A	A	T	G	NF	0.019	NF	0,015	0.039	NF	0.012
G	C	T	A	A	A	T	***C***	NF	NF	NF	0,013	NF	NF	0.016

The risk alleles in Europeans are in bold-face and italicized, and the core risk haplotype is marked with an asterisk. ^†^Population-specific haplotypes <5% frequent were omitted. NF, haplotype not found in given geographic region.

A handful of replication studies in non-European populations for CHD-related phenotypes have been published to date. Most of these studies make use of populations of East Asian ancestry [22-25] in which patterns of LD are similar to LD patterns in Europeans. Not surprisingly, these studies confirm previously described associations with disease phenotypes discovered in populations of European ancestry. A replication study in a multi-ethnic sample [26] that included relatively small numbers of cases and controls per ethnic origin confirmed association in Hispanics, but found no association in African Americans, possibly due to the small sample size and the low frequency of the alleles studied. Studies from populations of diverse ancestry are generally lacking. Our results demonstrate the importance of ancestry-specific allelic background when selecting SNPs for replication in global populations, and demonstrate that this approach can complement fine-mapping studies to possibly identify novel putative causative variant/s. Intriguingly, our data imply very different population histories for these two adjacent disease loci, with an increase in the prevalence of the T2D protective allele, most notably in East Asian populations, versus an increase in the prevalence of the CHD risk allele already in Middle Eastern populations. The HGDP SNP data we used here are publicly available and represent a valuable resource for studies of other complex diseases.

## Abbreviations

CEPH: Centre d'Etude du Polymorphisme Humain; CEU: HapMap data from 30 trios of Northern and Western European ancestry; CHD: coronary heart disease; HGDP: Human Genome Diversity Panel; LD: linkage disequilibrium; SNP: single nucleotide polymorphism; T2D: type 2 diabetes.

## Competing interests

The authors declare that they have no competing interests.

## Authors' contributions

LP and KS conceived of the study, and were in charge of study design and coordination. KS was in charge of the additional genotyping of markers on the Sequenom system, performed the bulk of the statistical analyses, and drafted the manuscript. SM and NT provided genotype data for SNP rs1333049, and SM helped in creating quality figures. HT and LCS provided the Illumina 650Y HGDP cleaned data and provided the HGDP DNA samples. HT and EJ were involved in the statistical analyses of the data. All authors participated in discussing study design and results interpretation, and read, commented and approved the final manuscript.

## Additional data files

The following additional data are available with the online version of this paper. Additional data file 1 is a Powerpoint file showing the pattern of linkage disequilibrium in the African HGDP samples on chromosome 9:22071397-22124172, a region approximately 53 kb long.

## Supplementary Material

Additional data file 1 R^2 ^values between each SNP pair are shown in shades of grey (black R^2 ^= 1, white R^2 ^= 0) and within each box. The SNPs best tagging the disease-associating haplotypes (rs4977574 and rs10811661) are in bold. The positions of two SNPs that have been identified as most strongly associated with CHD in two separate fine-mapping studies of Europeans, rs2891168 and rs10757278 (see main text), are shown above the genomic sequence line. The position of the *ANRIL *gene is shown in the upper panel, while the *CDKN2B *gene is located 72 kb upstream of the first SNP shown, rs10116277. Click here for file
