# Supplementary material for: Worldwide patterns of haplotype diversity at 9p21.3, a locus associated with type 2 diabetes and coronary heart disease
Source: Genome Med. 2009 May 12;1(5):51. doi: 10.1186/gm51 (PMC2689443; doi:10.1186/gm51)
Supplement: Additional data file 1 — R2 values between each SNP pair are shown in shades of grey (black R2 = 1, white R2 = 0) and within each box. The SNPs best tagging the disease-associating haplotypes (rs4977574 and rs10811661) are in bold. The positions of two SNPs that have been identified as most strongly associated with CHD in two separate fine-mapping studies of Europeans, rs2891168 and rs10757278 (see main text), are shown above the genomic sequence line. The position of the ANRIL gene is shown in the upper panel, while the CDKN2B gene is located 72 kb upstream of the first SNP shown, rs10116277. [file gm51-S1.ppt]

## Slide 1
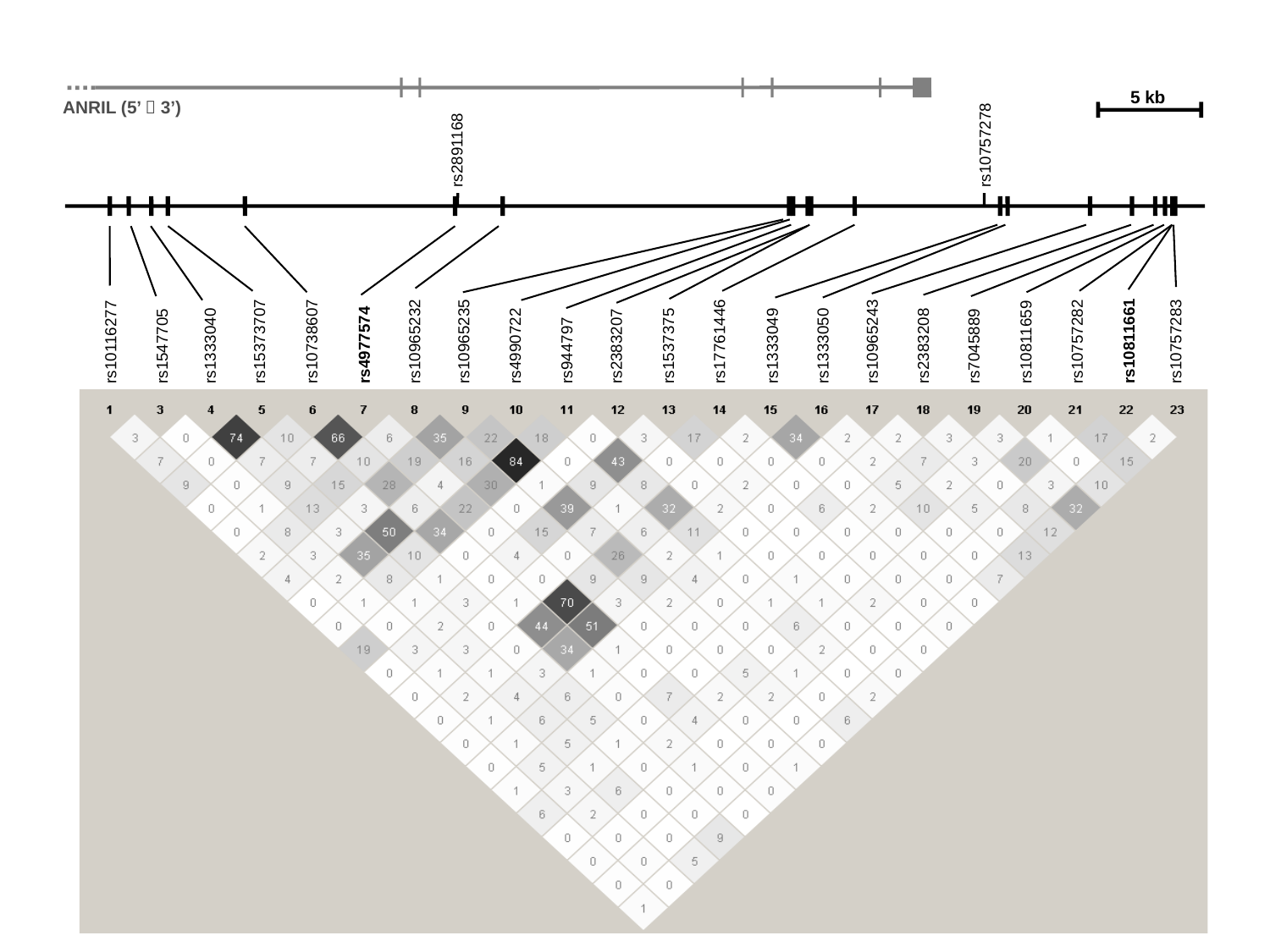

5 kb
ANRIL (5’  3’)
rs10757278
rs2891168
rs10116277
rs1333040
rs15373707
rs10738607
rs4977574
rs4990722
rs1537375
rs17761446
rs1333050
rs10965243
rs2383208
rs10811659
rs10811661
rs10757283
rs1547705
rs2383207
rs7045889
rs10757282
rs10965232
rs10965235
rs944797
rs1333049
T2D LD region
CHD LD region
